# Modulation of the PI3K/Akt/mTOR signaling pathway by probiotics as a fruitful target for orchestrating the immune response

**DOI:** 10.1080/19490976.2021.1886844

**Published:** 2021-02-21

**Authors:** Amir Hossein Mohseni, Vincenzo Casolaro, Luis G. Bermúdez-Humarán, Hossein Keyvani, Sedigheh Taghinezhad-S

**Affiliations:** a Department of Microbiology, Faculty of Basic Sciences, Science and Research Branch, Islamic Azad University, Tehran, Iran; b Department of Medicine, Surgery and Dentistry “Scuola Medica Salernitana”, University of Salerno, Baronissi, Salerno, Italy; c Université Paris-Saclay, INRAE, AgroParisTech, Micalis Institute, Jouy-en-Josas, France; d Department of Virology, Faculty of Medicine, Iran University of Medical Sciences, Tehran, Iran

**Keywords:** PI3K, akt, mTOR, probiotics, *lactobacillus*, *bifidobacterium*

## Abstract

The mammalian target of rapamycin (mTOR) and the phosphatidylinositol-3-kinase (PI3K)/protein kinase B or Akt (PKB/Akt) signaling pathways are considered as two but somewhat interconnected significant immune pathways which play complex roles in a variety of physiological processes as well as pathological conditions. Aberrant activation of PI3K/Akt/mTOR signaling pathways has been reported to be associated in a wide variety of human diseases. Over the past few years, growing evidence in *in vitro* and *in vivo* models suggest that this sophisticated and subtle cascade mediates the orchestration of the immune response in health and disease through exposure to probiotics. An expanding body of literature has highlighted the contribution of probiotics and PI3K/Akt/mTOR signaling pathways in gastrointestinal disorders, metabolic syndrome, skin diseases, allergy, salmonella infection, and aging. However, longitudinal human studies are possibly required to verify more conclusively whether the investigational tools used to understand the regulation of these pathways might provide effective approaches in the prevention and treatment of various disorders. In this Review, we summarize the experimental evidence from recent peer-reviewed studies and provide a brief overview of the causal relationship between the effects of probiotics and their metabolites on the components of PI3K/Akt/mTOR signaling pathways and human disease.

## Introduction

Probiotics are live microorganisms which when administered in adequate amounts confer a health benefit on the host.^1^ Decades of research have clearly demonstrated the beneficial effects of probiotics. These effects include improvement of gastrointestinal health and gut immunity, prevention of potential colonization by pathogenic bacteria and reducing the risk of certain type of cancers.^2–5^ Most probiotic bacteria belong to the genus *Lactobacillus, Bifidobacterium, Lactococcus*, and *Enterococcus* spp., and these microorganisms offer valuable alternative approaches for future immunomodulatory and cancer prevention therapies.^6–9^

Protein kinase B, also known as Akt (PKB/Akt), a serine/threonine protein kinase and a direct downstream effector of phosphoinositide 3-kinase (PI3K), is a key component of the PI3K/mechanistic target of rapamycin (mTOR)/Akt signaling network.^10,11^ mTOR, the target molecule of rapamycin, is a serine/threonine kinase located downstream of the PI3K/Akt pathway.^12^ The mTOR signaling cascade involves two multiprotein complexes with different functions, mTORC1 and mTORC2.^13^ This is highlighted in extensive studies showing that the mTOR signaling pathway has apparent regulatory impact on immune function and T-cell differentiation by integrating various micro-environmental signals.^14,15^ Detailed investigations of the mTORC signaling pathway showed that activation of mTORC1 follows activation of PI3K, which in turn, by different interactions with pyruvate dehydrogenase kinase 1 (PDK1), can phosphorylate and partially activate Akt at Threonine (Thr)-308.^16^ Moreover, subsequent phosphorylation of Akt at Serine (Ser)-473 by mTORC2 leads to its full activation and impact on regulation of stress resistance, glucose metabolism, apoptosis, and cell proliferation through blocking of transcription factors forkhead box O1 (FOXO1)/3a.^17,18^ It has been demonstrated that up-regulation of Akt is followed by the activation of the lipid kinase PI3K via RAS guanosine triphosphate-binding proteins (GTPases).^19^ Additionally, several evidences indicate that Akt can indirectly promote the activation of ribosomal S6 kinase (S6K) and eukaryotic translation initiation factor 4E (eIF4E)-binding protein 1 (4E-BP1) through direct phosphorylation of mTOR.^11,20^ Studies in different models clarify the role of mTOR signaling pathway in the regulation of cell cycle via the PI3K/Akt/mTOR/S6K cascade.^21,22^ Since the PI3K/Akt/mTOR signaling pathway is extensively reviewed elsewhere, additional comprehensive description may be found in other reviews.^23–25^ However, studies in the past few years have made progressively clear that the PI3K/Akt/mTOR cascade can respond to different stimuli to regulate signaling pathways and essential processes of cellular biology, including crucial processes such as growth, survival, proliferation, and cell metabolism that are dysregulated in different disorders.^23,26^ Aberrant activation of the PI3K/Akt/mTOR network contributes to pathological conditions, including type 2 diabetes (T2D), nonalcoholic fatty liver disease (NAFLD), and cancer. Therefore, modulating the components of the PI3K/Akt/mTOR pathway has recently been proposed as a vital and potential therapeutic option for preventing and/or treating a quite diverse host of conditions whose chronic complications are important burdens in modern communities.^17,27,28^

**Figure 1.**
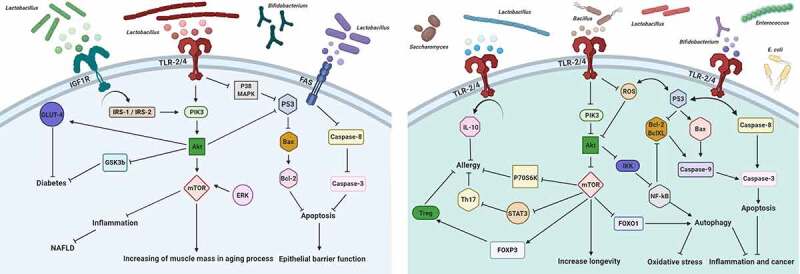
Schematic representation of the complex crosstalk between probiotics and the PI3K/AKT/mTOR signaling network, whereby extracellular and intracellular signals converge to orchestrate canonical upstream and downstream pathways to modulate a wide range of biological processes involved in various disorders. A detailed description of these interactions is provided in the text. Right: Up-regulation of PI3K/AKT/mTOR signaling cascade by probiotics. Left: Down-regulation of hyperactivated PI3K/AKT/mTOR signaling pathway by probiotics. Arrows indicate positive regulation (activation/stimulation), bar-headed lines indicate negative regulation (inhibition). The abbreviations shown in the figure can be found in the main text. (Figure was designed by https://biorender.com)

Within the last decade, the fields of immunology and microbiology have turned out to be more entwined than previously thought. Up until now, considerable effort and an ever-growing number of *in vitro* and *in vivo* studies have been conducted to investigate the possible intriguing link between the effects of probiotics on the PI3K/Akt/mTOR signaling network and their effects on different aspects of health and disease. Major efforts in recent years have been made to shed light on the mechanisms accounting for the interconnections between probiotics metabolism and PI3K/Akt/mTOR signaling pathway (Table 1). For example, several findings document a possible relationship between the consumption of probiotic strains and their derived metabolites, and the downregulation of mTOR signaling, resulting in improvement of allergic responses.^29^ In line with this hypothesis, an important *in vivo* study conducted by Jeong *et al*., looking at the effectiveness of *Lactobacillus plantarum* KY1032 in controlling lipidemia in rats, discovered that the oral consumption of this probiotic can result in concomitant reduction of Akt and mTOR phosphorylation.^33^ Similarly, the suppressing effect of *Lactobacillus rhamnosus* GG (LGG) strain on the phosphorylation levels of Akt/mTOR and extracellular-signal regulated kinases1/2 (ERK1/2) has been demonstrated in parallel with symptom improvement in animal models of obstructive sleep apnea (OSA).^45^ Keeping these results in mind, probiotic strains should be selected based on their ability to regulate PI3K/Akt/mTOR signaling intermediates for targeted therapy of diseases in which activation of this pathway plays a role. In this Review, we wish to provide the Reader an exhaustive overview of how such studies have contributed to improve our understanding of the causal relationships between probiotics and the PI3K/Akt/mTOR signaling pathway (Figure 1). We herein summarize our current understanding of the modulatory effects of probiotics on the PI3K/Akt/mTOR signaling pathway in specific disease models to help pave the way for novel therapeutic strategies.

**Table 1.** List of probiotics strain and their effect on different disorders

Probiotic	Model	Mechanism	Effect	Year	Reference
*B. coagulans* 09.712	*In vivo*	Up-regulation of Foxp3 and down-regulation the phosphorylation of Akt, 4E-BP1, STAT3, SGK1, p70S6K and mTOR	Amelioration of allergic inflammation	2017	29
*L. plantarum*	*In vitro*	Up-regulation of PTEN, BAX, TLR4, and down-regulation of Akt genes	Induction of apoptosis and inhibition of H. pylori-related gastric cancer	2020	30
	*In vivo*	Reduction of lipid accumulation, FASN expression, and hepatic weight, and elevation of IRS-1/AKT/eNOS pathway expression and phosphorylation	Improvement of lipogenesis and restoring hepatic and renal dysregulation	2020	31
	*In vitro*	Inhibition of melanogenic enzymes and cellular activity of tyrosinase, and activation of PI3K/Akt and ERK pathways	Prevention of melanogenesis	2015	32
*L. plantarum* KY1032	*In vivo*	Inhibition of Akt/mTOR/NF-κB phosphorylation and activation of p53, p16, and COX-2 expression	Returning the alternation of age reduced spontaneous and amelioration of lipidemia	2015	33
*L. plantarum* WCFS1	*In vitro* and *in vivo*	Up-regulation of PKC and PI3K/Akt anti-apoptotic pathways	Regulation of TJ and actin cytoskeleton, and promotion of general immune tolerance	2021	34
*L. plantarum* H31	*In vitro*	Up-regulation of Akt-2, AMPK, and GLUT-4 expression	Prevention of pancreas α-amylase activity, and alleviation of diabetes mellitus	2020	35
*S. cerevisiae*	*In vitro*	Down-regulation of *p*-Akt1, Bcl-XL, pro-caspase-3 and 9 expressions, and up-regulation of BAX, cleavedcaspase-3 and 9	Induction of apoptosis in colon cancer	2020	36
*B. amyloliquefaciens* SC06	*In vitro* and *in vivo*	Inhibition of PI3K/Akt pathway	Alleviation of oxidative stress	2019	37
*B. licheniformis* SC08	*In vitro* and *in vivo*	Inhibition of PI3K/Akt pathway	Alleviation of oxidative stress	2019	37
*E. faecalis*	*In vitro*	Inhibition of Akt and mTOR phosphorylation	Induction macrophages autophagy	2018	38
*L. fermentum*	*In vitro*	Reduction of senescence markers, NF-κB activation and DNA damage, and down-regulating the phosphorylation of PI3K/Akt/mTOR pathway	Prevention of senescence progression	2020	39
*L. fermentum* L930BB	*In vitro* and *in vivo*	Up-regulation of PKC and PI3K/Akt anti-apoptotic pathways	Regulation of TJ and actin cytoskeleton, and promotion of general immune tolerance	2021,2018	34,40
*L. rhamnosus*	*In vivo*	Down-regulation of PI3K/mTOR/NF-κB pathways	Reduction of autophagy and inflammation, and improvement of alcoholic hepatitis	2019	41
*L. rhamnosus* GG	*In vitro* and *In vivo*	Down-regulation of p38 MAPK and up-regulation of PI3K/Akt cascade	Prevention of cytokine-induced apoptosis	2007	42
	*In vivo*	Enhancing mTOR signaling pathway expression, antioxidant activities and tight junction, and attenuating apoptosis and autophagy	Protection of LPS-induced intestinal barrier dysfunction	2017	43
	*In vitro*	Suppression of PI3K/Akt signal pathway over-activation	Regulation of Salmonella induced IL-8 response and prevention of Salmonella infection	2016	44
	*In vivo*	Suppression of Akt/mTOR and ERK1/2 pathways phosphorylation	Improvement of OSA	2019	45
*L. acidophilus* NCFM	*In vivo*	Down-regulation of Akt1, p38 and cytosolic group IV PLA2 phosphorylation, and up-regulation of ERK1/2 phosphorylation	Reduction of T-cell-induced colitis	2011	46
	*In vitro*	Down-regulation of JNK and mTOR pathways, and up-regulation of p53 and p21 proteins expression, and ROS formation	Induction of autophagic cell death and anti-cancer effect	2019	47
*K. marxianus*	*In vitro*	Prevention of mTOR, JAK-1, and Akt-1 pathways	Induction of apoptosis	2020	48
*P. kudriavzevii*	*In vitro*	Prevention of mTOR, JAK-1, and Akt-1 pathways	Induction of apoptosis	2020	48
*L. reuteri*	*In vivo*	Elevation of active-β-catenin and TGFβ1 expression, and PI3K/Akt phosphorylation	Stimulation of GMSCs function and healing of wound	2019	49
*L. reuteri* ZJ617	*In vivo*	Enhancing mTOR signaling pathway expression, antioxidant activities and tight junction, and attenuating apoptosis and autophagy	Protection of LPS-induced intestinal barrier dysfunction	2017	43
*L. reuteri* GMNL-263	*In vivo*	Down-regulation of Fas ligand and up-regulation of IGF-IR/PI3K/Akt cell survival pathway	Reduction of hyperlipidaemic and cardiac apoptosis	2015	50
*B. bifidum*	*In vitro*	Inhibition of LPS-induced autophagy and punctate distribution of GFP-mCherry-LC3, and decreasing of LC3-II/LC3-I ratio	Maintaining epithelial barrier function	2016	51
*C. butyricum*	*In vivo*	Up-regulation of TJ-related proteins expression, and Akt/mTOR, and p70S6k phosphorylation	Reduction of intestinal mucosal permeability, and colitis symptoms	2020	20
	*In vivo*	Increasing the levels of Bcl-2, BDN, and Akt phosphorylation and decreasing the level of BAX	Reduction of neuronal apoptosis and improvement of VaD	2015	52
*C. butyricum* MIYAIRI 588	*In vitro* and *in vivo*	Reduction of hepatic lipid deposition and improvement of triglyceride, insulin resistance, and hepatic inflammatory indexes, and elevation of lipolysis or lipogenesis, Akt, and hepatic AMPK expression	Prevention of NAFLD progression	2013	53
*L. gasseri* JM1	*In vitro*	Activation of the TLR2 and NOD2-mediated PI3K/Akt signaling pathway	Alleviation of inflammation	2020	54
*L. paragasseri* K7	*In vitro* and *in vivo*	Up-regulation of PKC and PI3K/Akt anti-apoptotic pathways	Regulation of TJ and actin cytoskeleton, and promotion of general immune tolerance	2020	34
*B. lactis* Bb-12	*In vivo*	up-regulation of insulin receptor substrate 1 and insulin receptor beta, Akt, certain adipocytokines, IκBα, and IKKα expression, and down-regulation of mTOR and p66Shc signaling pathway	Improve glucose homeostasis	2018	55
*B. animalis* subsp. *lactis* BI-04	*In vitro*	Up-regulation of PI3K/Akt pathways and down-regulation of p53 gene expression	Postpone the BaP-induced apoptosis	2020	56
*B. animalis* subsp. *lactis* DSM10140	*In vitro*	Suppression of PI3K/Akt signal pathway over-activation	Regulation of Salmonella induced IL-8 response and prevention of Salmonella infection	2016	44
*B. animalis* subsp. *animalis* (IM386)	*In vitro* and *in vivo*	Up-regulation of PKC and PI3K/Akt anti-apoptotic pathways	Regulation of TJ and actin cytoskeleton, and promotion of general immune tolerance	2021	34
*B. breve* B-3	*In vivo*	Promotion of AMPK and Akt/mTOR signaling phosphorylation	Elevation muscle mass	2020	57
*B. breve*C50 (BbC50sn)	*In vitro*	Induction of PI3K/Akt, p38MAPK, and ERK pathways	reduction of allergic and inflammatory disorders	2008	58
*L. johnsonii* N6.2	*In vivo*	Reduction of mTORC1-activating phosphorylation of pAKT-T308 and pAKT-S473	Reduction of metabolic syndrome disease	2018	59
*L. casei*	*In vitro*	Down-regulation of PI3K/Akt/NF-κB phosphorylation	Induction of apoptosis and inhibition of gastric cancer	2013	60
*L. casei* Zhang (LCZ)	*In vivo*	Decreasing inflammatory cytokines, and inhibiting the hyperactivation of PI3K/Akt/NF-κB and *p*-STAT3 pathways	Prevention and treatment of ulcerative colitis	2019	95
*L. paracasei* subp. *paracasei* X12	*In vitro*	Down-regulation mTOR/4E-BP1 pathway and cyclin E_1_, and up-regulating of p27	Blocking cell cycle of colon cancer cells	2016	61
*L. paracasei* TD062	*In vitro* and *in vivo*	Increasing the level of IRS-2, PI3K and Akt and decreasing the level of GSK-3β	Improvement of glucose homeostasis and insulin resistance, and inhibition of T2DM	2018	62
VSL#3	*In vivo*	Inhibiting the PI3K/Akt and NF- κB pathway, iNOS, COX-2, NF-κB, TNF-α, IL-6, and *p*-Akt expression, and increasing of IL-10	Prevention and treatment of ulcerative colitis	2013	63
*L. mesenteroides*	*In vitro*	Inactivation of NF-κB, Akt, and Bcl-XL expressions, and up-regulation of MAPK1, BAX, and caspase-3	Promotion of apoptosis in colon cancer	2017	64
*L. salivarius* Ren	*In vitro* and *in vivo*	Inactivation of Akt signaling cascades, cyclinD_1_, and COX-2	Suppression of cell proliferation and CRC formation, and induction of cell apoptosis	2020	65
*L. salivarius* AR809	*In vivo*	Inhibition of TLR/PI3K/Akt/mTOR/NF-κB pathway, and elevation of autophagic protein level	Inhibition of inflammatory response caused by *S.aureus*	2020	66
*L. curvatus* HY7601	*In vivo*	Inhibition of Akt/mTOR/NF-κB phosphorylation and activation and p53, p16, and COX-2 expression	Returning the alternation of age reduced spontaneous	2015	33
*L. pentosus* var. *plantarum* C29	*In vivo*	Suppression of p16, COX-2, and inducible nitric oxide synthase expression, and activation of Akt, mTOR, and NF-κB	Returning the alternation of age reduced spontaneous	2015	67
*E. faecium* L-15	*In vivo*	Activation of PI3K/Akt pathway	Improvement of self-renewal and proliferation of SKPs	2020	6

## Probiotics, autophagy, and apoptosis

Autophagy, or programmed cell death (PCD) type 2, an evolutionarily conserved pathway, is a lysosome-mediated catabolic pathway and plays a critical role in degradation of the organelles and superfluous proteins that happens ubiquitously in all eukaryotic cells.^68^ It has recently been highlighted as an innate defense or vital homeostasis mechanism against bacterial pathogens and a variety of stimuli and metabolic stress conditions, including nutrient deprivation, which is fundamental for cytoplasmic recycling, cellular bioenergy homeostasis, cellular lipid metabolism, cell survival, and lifespan extension.^69^ Studies in animal models and in humans are starting to unravel the opposing link between autophagy and inflammation. Of note, the inflammatory response mediated by the nuclear factor kappa B (NF-κB) signaling cascade would result in inhibition of autophagy, while, conversely, an inflammatory response would be attenuated after activation of autophagy.^70^ Within the past years, it has become increasingly clear that several key molecular and signaling pathways play a crucial role in regulating the balance of autophagy *vs*. non-apoptotic cell death. Among the most studies are the PI3K/Akt/mTOR signaling pathway, adenosine 50-monophosphate (AMP)-activated protein kinase (AMPK), and mitogen-activated protein kinase (MAPK)/ERK signaling pathways, which have the capability to regulate autophagy at diverse steps of autophagosome formation.^71,72^ In cancerous cells, in response to cellular stress induced by chemotherapeutics, mTOR has the potential to regulate the balance between autophagy and cell proliferation.^73^ Nevertheless, in the presence of growth factors or nutrients, autophagy would be down-regulated following the activation of TORC1 and TORC2 by PI3K-I (class I PI3Ks) and Akt, and the phosphorylation of their downstream molecules.^74^ In contrast, in the absence of nutrients and growth factors and/or the presence of other stressors, autophagy will be up-regulated due to the inhibition of Akt/mTOR activation.^75^ The observation that there is negative cross-regulation between autophagy and the PI3K/Akt/mTOR signaling pathway can have interesting implications in the regulation of cellular lipid metabolism.^76^ Recent experimental evidence gathered from animal models characterized the effects of probiotics on PI3K/Akt/mTOR signaling, autophagy, and indicators of inflammation. All in all, such studies highlighted a novel mechanism and a theoretical foundation for the inhibitory effects of probiotics on expression of the pro-inflammatory cytokines, IL-1β, IL-6, and TNF-α, and their association with reduced mTOR/FOXO1/NF-κB activity and the promotion of autophagy processes in normal cellular lipid biosynthesis.^77^ These observations have been documented by both *in vitro* and *in vivo* studies which have probed that some strains of probiotics (*e.g. Bacillus amyloliquefaciens* SC06 and *Bacillus licheniformis* SC08) could improve oxidative stress by promoting the intestinal autophagy machinery system following inhibition of the PI3K/Akt pathway.^37^

Aging is a multi-factorial deleterious process that accounts for increased morbidity and mortality in elderly and is accentuated in certain disease states, especially in people living with HIV (PLWH). Increasing evidence indicates that the gathering of damaged cellular components related to the aging process, due to the accumulation of reactive oxygen species (ROS), contributes to dysregulated autophagy. Remarkably, increased activity of the PI3K/Akt axis, together with inhibition of autophagy, are a causative node in several diseases attributed to the aging process, including type 2 diabetes mellitus (T2DM), neurodegeneration, cancer, and heart disease. During aging, skeletal muscle mass could be reduced due to alterations in the activity of autophagy.^78,79^ In this context, studies have documented the unique role of probiotics-induced mTORC signaling in cytoskeletal organization. In fact, increased skeletal muscle mass has been demonstrated in rats administered heat-killed *Bifidobacterium breve* B-3, possibly mediated by increased phosphorylation of Akt/mTOR signaling intermediates along with AMPK.^57^ It is well known that cellular senescence plays an essential role in the aging process. In recent years, it has been demonstrated that the PI3K/Akt/mTOR cascade critically regulates various processes associated with cell senescence during aging, and can be successfully engaged to extend the longevity in aged mice and humans by improving immune function.^80^ Striking results from animal models support the pro-longevity effects of probiotics. For example, administration of the secretory metabolites of *Lactobacillus fermentum* in mice models of senescence can effectively attenuate the development and progression of senescence by decreasing senescence markers such as p21WAF1, p53, p38MAPK, ROS, cyclooxygenase-2 (COX-2), inducible nitric oxide synthase (iNOS), and senescence-associated-β-galactosidase (SA-β-gal), by inhibiting NF-κB activation and the DNA damage response, and by down-regulating the phosphorylation of PI3K/Akt/mTOR signaling intermediates.^39^ Similar results were seen in aged Fischer 344 rats administered *Lactobacillus plantarum* KY1032, *Lactobacillus curvatus* HY7601, and *Lactobacillus pentosus* var. *plantarum* C29, in which these probiotics caused inhibition of the phosphorylation and activation of Akt/mTOR/NF-κB pathway intermediates and expression of p53, p16, and COX-2, and returned alternation of age reduced spontaneous in aged rats.^33,67^ Additional approaches will be essential to elucidate the interactions of probiotics with the PI3K/Akt/mTOR signaling pathway in the regulation of aging processes.

Apoptosis is another important form of PCD. A numbers of excellent studies have shown that the intrinsic and extrinsic apoptosis pathways play vital roles in tumor regression and are in turn regulated by the PI3K/Akt, p38 MAPK, c-Jun N-terminal kinase (JNK), and AMPK pathways.^81,82^ It has been shown that *Lactobacillus rhamnosus GG* and its metabolites could prevent cytokine-induced apoptosis in human or mouse intestinal epithelial cells by down-regulating the activation of p38 MAPK and up-regulating the PI3K/Akt cascade.^42^ Both live and dead cells of *Bifidobacterium animalis* subsp. *lactis* BI-04 strain can retard Benzo(a)pyrene (BaP)-induced apoptosis of the colonic epithelial cells by up-regulating the PI3K/Akt signaling pathway and down-regulating p53 gene expression.^56^ In light of these findings, some researchers have hypothesized that the induction of apoptosis in the SW480 cell line could occur in the presence of heat-killed preparations of *Saccharomyces cerevisiae* through enhanced expression of BAX, cleaved caspase-3, and cleaved caspase-9 and reduced expression of *p*-Akt1, Bcl-XL, pro-caspase 3 and 9, which are involved in the Akt/NF-κB signaling pathway.^36^ Therefore, by targeting apoptosis and survival-related signaling pathways these probiotics may offer important therapeutic options for cancer management.

Ample evidence indicates a critical role for vascular events resulting in impaired blood flow and blood vessel damage in the brain in the development of vascular dementia (VaD). VaD is implicated in behavioral deterioration, progressive cognitive, memory and learning deficits, associated with neurodegeneration and neural lesions. Conclusive findings linking neuronal apoptosis to a low ratio of Bcl-2/BAX have been obtained in studies of hippocampal lesions in rat models of VaD. Conversely, it has been shown that the up-regulation of PI3K/Akt pathway can rescue neurons from apoptosis activated in cerebral ischemia-reperfusion (IR) injury.^83,84^ Recent studies highlight the potential role of Akt/mTOR signaling in the prevention of vascular cognitive damage by probiotics. These observations indicate that probiotics such as *Clostridium butyricum* may prevent VaD by increasing the levels of Bcl-2, BDN, and Akt phosphorylation and decreasing the levels of BAX, leading to reduced neuronal apoptosis and improvement of cognitive function in bilateral common carotid artery occlusion (BCCAO)-induced VaD. Taken together, the aforementioned data strongly point to the modulation of apoptosis, via the regulation of Bcl-2/BAX ratio, as a possible therapeutic target in VaD.^52^

## Probiotics and their surface components

Emerging studies have demonstrated that the anti-inflammatory immune mechanisms and anti-tumoral effects of probiotic are closely associated with their surface components. This has prompted investigators to look at the key components of the cell surface of probiotics such as lipoteichoic acid (LTA), surface-layer protein (Slp), and exopolysaccharides (EPS).^85^ LTA, an amphiphilic negatively charged glycolipid, is an immune-stimulatory component of the cell wall of probiotics that promotes the attachment of probiotics to host cells, colonization, and invasion.^46^ At present, there are conflicting findings in the sparse literature on the role of LTA in the regulation of inflammation. In some of these studies, LTA appears to play an important role in severe inflammatory responses and the pathogenesis of septic shock by stimulating the production of cytokines, such as IL-6 and TNF-α, via toll-like receptors (TLRs) such as TLR2.^46,86^ The stimulatory role of LTA in the development of septic shock is supported by *in vivo* evidence in a mouse model in which oral administration of *Lactobacillus acidophilus* strain NCFM deficient in LTA (NCK2025), compared to administration of the wild-type parental strain (NCK56), can down-regulate the phosphorylation of Akt1, p38 and cytosolic group IV PLA2 in colonic epithelial cells and dendritic cells, but significantly augment phosphorylation of ERK1/2, which highlights the overall immunosuppressive effect of NCK2025 in T-cell-induced colitis.^46^ However, in apparent conflict with these findings, data strongly suggest that LTA can play a major role in the generation of anti-inflammatory cytokines, such as IL-10, and in maintaining intestinal cell homeostasis through up-regulation of ERK1/2 signaling.^32,87^ These opposing effects are important caveats when trying to envisage and/or interpret the aftereffects of probiotics treatment. As of today, the functional properties of LTA are under intensive scrutiny in *in vivo* studies to fully elucidate its overall effects on innate immunity. It has long been known that inflammation can be promoted by macrophages through the production of different cytokines, concomitant with the uptake and eradication of pathogens.^46,88^ Regarding this issue, it is becoming increasingly clear that probiotics can sustain the immune response by promoting autophagy in pathogen-loaded macrophages. It is generally accepted that *Enterococcus faecalis* LTA can stimulate macrophages autophagy *in vitro* via hindering the phosphorylation of mTOR and Akt.^38^ The molecular mechanism underlying the anti-tumoral properties of Slp derived from *Lactobacillus acidophilus* NCFM were reported in HCT116 cells. These data support the notion that Slp can induce autophagic cell death following inhibition of cell proliferation by controlling the JNK and mTOR signaling pathways via reducing the phosphorylation levels of 4E-BP1, p70, and S6, and by up-regulating the expression of p53 and p21, and generation of ROS.^47^ It has been established that, in addition to *Lactobacillus, Kluyveromyces marxianus* and *Pichia kudriavzevii* fulfill the major criteria for probiotics definition. Of note, also the EPSs of these strains can induce apoptosis and may treat CRC by interfering with the mTOR, Janus Kinase 1 (JAK-1), and Akt-1 pathways.^48^

## Probiotics and Gastrointestinal (GI) disorders

Disruption in barrier function and alterations in tight junctions (TJ) structure are linked to the development of chronic inflammation. These changes may lead to various complications, including immune cell infiltration, expression of inflammatory cytokines, translocation of bacteria, and finally stimulation of systemic inflammatory responses.^89^ Several findings also suggest that mechanisms controlling cell apoptosis and proliferation, that is, epithelial turnover, are deeply implicated in preserving intestinal integrity, whereby an augmented rate of apoptosis in epithelial cells is associated with injury of the intestinal mucosa.^43,90,91^ This is highlighted in extensive studies showing that an impairment of epithelial barrier function and reduced synthesis of TJ-associated proteins were subsequent to *p*-S6K and *p*-mTOR down-regulation in response to reduced Akt and *p*-Akt protein expression in dextran sulfate sodium (DSS)-treated mice.^20,40^ Studies conducted over the last few years have contributed to our understanding of the causal association between the activation of the PI3K/Akt/mTOR signaling pathway, the induction of TJ-associated proteins and promotion of intestinal epithelial barrier function. Given the potential connection between gut microbiota and GI inflammatory disorders, several authors have been prompted to investigate the properties of beneficial microbes in experimental models of inflammation to identify new possible approaches to manage these conditions. It was long believed that *Lactobacillus* strains have the ability to prevent many human diseases and cancer development, and enhance the production of anti-inflammatory cytokines involved in innate immunity through the modulation of the PI3K/Akt/mTOR pathway, given its involvement in various cellular processes, such as apoptosis, inflammatory responses, and tumor angiogenesis.^63^ Subsequent studies revealed that epithelial barrier function both in *in vitro* and *in vivo* could be markedly improved by pretreatment with such probiotics as *Bifidobacterium bifidum, Lactobacillus reuteri*, and LGG and its proteins (p75 and p40).^43,51,92^ Similar results were seen in mice models, where ingestion of LGG and *Lactobacillus reuteri* ZJ617, by reducing autophagy and apoptosis via the activation of the mTOR cascades, and therefore improving TJ integrity, hinder lipopolysaccharides (LPS)-stimulated intestinal barrier dysfunction.^43^ This view is also supported to a certain extent in studies showing that *Clostridium butyricum* can contribute to improving the intestinal barrier function in a mouse model of DSS-induced colitis. The above findings provided a basis to further explore the underlying mechanism of these beneficial properties of *Clostridium butyricum*, which was found to increase the expression of TJ-associated proteins and diminish intestinal mucosal permeability via the up-regulation of the Akt/mTOR axis and the phosphorylation of their downstream signaling molecules such as p70S6k.^20^ Accumulating *in vitro* and *in vivo* evidence indicates that such probiotic strains as *Lactobacillus fermentum* L930BB, *Lactobacillus paragasseri* K7, *Bifidobacterium animalis* subsp. *animalis* (IM386), and *Lactobacillus plantarum* WCFS1, via the up-regulation of protein kinase C (PKC) and PI3K/Akt anti-apoptotic pathways, can regulate the actin cytoskeleton and TJ structure to ensure reconstitution of the intestinal epithelial barrier.^34,40^ Most importantly, the PI3K/Akt/mTOR network complex, via cooperation with TLR-delivered signals and NF-κB, plays fundamental roles in the development of inflammatory responses. In this context, it is documented that *Lactobacillus gasseri* JM1 could improve inflammation in Caco-2 cells treated with LPS by up-regulation of TLR2 and nucleotide-binding oligomerization domain containing 2 (NOD2)-mediated PI3K/Akt network.^54^

Dysregulation and hyperactivation of the PI3K/Akt/mTOR signaling network are closely associated with cell proliferation, resistance to apoptosis, and metastasis formation, and ultimately contribute to the progression of inflammatory bowel disease (IBD), colorectal cancer (CRC), and gastric cancer.^93,94^ Additional studies have addressed how the PI3K/Akt/mTOR signaling pathway can mediate control of colon and gastric cancer development by probiotics. In keeping with this, studies in murine models have demonstrated that *Lactobacillus casei* Zhang (LCZ) and VSL#3 could prevent and treat ulcerative colitis by decreasing the production of host inflammatory cytokines, inhibiting the hyperactivation of the PI3K/Akt/NF-κB pathways and the phosphorylation of signal transducer and activator of transcription 3 (STAT3).^63,95^ Well-designed studies in murine models have also shown that administration of *Leuconostoc mesenteroides* and *Lactobacillus salivarius* Ren could significantly induce cell apoptosis in colon cancer cell lines and block colon cancer progression. The proposed mechanisms underlying the anti-tumoral effects of these strains included inactivation of the NF-κB and Akt signaling cascades and their downstream molecules (cyclin D_1_ and COX-2), down-regulation of anti-apoptotic protein like Bcl-XL and Bcl-2, and up-regulation of MAPK1, caspase 3 and 8, and BAX.^64,65^ In light of these findings, it is worth mentioning that *Lactobacillus paracasei* such as species X12 can block the G1 phase of human colorectal adenocarcinoma cell cycle by down-regulating the mTOR/4E-BP1 signaling pathway through the up-regulation of p27 and the down-regulation of cyclin E_1._^61^ Similarly reduced phosphorylation of PI3K/Akt/NF-κB signaling intermediates by probiotics was later confirmed in gastric cancer cells by Hwang and colleagues. They showed that *Lactobacillus casei* extracts can up-regulate apoptosis in gastric cancer cells and prevent gastric cancer by down-regulating the phosphorylation of PI3K/Akt/NF-κB signaling components.^60^ As shown in new report, *Lactobacillus plantarum* can inhibit both *Helicobacter pylori* colonization and a gastric cancer cell line (AGS) through the up-regulation of PTEN, BAX, TLR4, and the down-regulation of Akt genes.^30^ Hence, blocking the hyperactivation of the PI3K/Akt/mTOR signaling pathway has emerged as a plausible therapeutic target for CRC and gastric cancers because of its involvement in cell growth and proliferation. However, the mechanisms of action involved in the beneficial effects of probiotics mediated through the PI3K/Akt/mTOR signaling pathway in GI disorders are not fully elucidated in relevant human models. Thus, determining the exact mechanisms of action is not only crucial to determining the pathogenesis of GI disorders, but would also provide a new basis for emerging therapies. To this end, prospective clinical trials should be launched to better characterize patients diagnosed with GI disorders.

## Probiotics and Metabolic syndrome

The metabolic syndrome (MetS) is defined by the coexistence of risk factors such as increased abdominal fat, obesity, high blood sugar, high blood pressure, and hyperlipidemia that predispose to the development of T2D, cardiovascular disease (CVD), and NAFLD.^96^ As indicated earlier, over-activation of the mTOR pathway is associated with the process of autophagy, leading to several metabolic disorders.^17^ Given the possible connection between probiotics and MetS, many authors have also been encouraged to investigate the effects of probiotics on PI3K/Akt signaling and MetS development in murine models. The following paragraphs provide an overview of the probiotics investigated in studies of the PI3K/Akt/mTOR signaling pathway and of their effects on MetS progression.

### Type 2 diabetes (T2D)

T2D is characterized by disordered glucose metabolism as a result of insulin resistance. Attachment of insulin to insulin receptor substrate 2 (IRS-2) results in phosphorylation of PI3K and Akt, which improves glucose metabolism by phosphorylating glycogen synthase kinase 3 beta (GSK-3β). Conversely, blocking the activity of the PI3K/Akt signaling pathway following the inhibition of IRS-2 phosphorylation may cause high glucose concentrations because of the hyperphosphorylation of GSK-3β.^97^ A mounting body of evidence indicates that this dysfunction could be reversed in the presence of probiotic microorganisms. For example, oral administration of *Lactobacillus paracasei* TD062 to diabetic mice could ameliorate the insulin response and glucose homeostasis via decreasing the levels of GSK-3β and elevating those of IRS-2, PI3K and Akt, thus preventing the development of T2DM.^62^

Two significant factors contributing to lipogenesis and insulin resistance are life-span determinant p66Shc (a 66 kDa proto-oncogene Src homologous-collagen homolog (Shc) adaptor protein) and the mTOR/S6K cascades. Data so far collected indicate that dysregulation in insulin signaling pathway, insulin resistance in muscle and liver tissues and lipid accumulation occurs following aberrant up-regulation of mTOR signaling. In particular, over-activation of mTORC1 and mTORC2 results in insulin resistance and gluconeogenesis suppression by up-regulation of S6K1 and Akt signaling, respectively. These events are followed by deposition of extra fat in the liver, hindering insulin signaling, and activation of glycolysis.^98,99^ *In vivo* studies observed that *Bifidobacterium* spp. such as *Bifidobacterium lactis* Bb-12 can reduce blood glucose levels by up-regulating the expressions of proteins involving in the insulin signaling pathway like insulin receptor substrate 1 and insulin receptor beta, down-regulating the excess activation of the mTOR and p66Shc pathways, and increasing the expression of certain adipokines, nuclear factor-kappa B inhibitor alpha (IκBα), and IκB kinase alpha (IKKα).^55,100^

It has been known for long time that the pancreas is the only tissue that secretes insulin and has a significant role in regulating glucose metabolism. Recent *in vivo* findings reveal that protection of the pancreas from β-cell apoptosis is mediated by probiotics via induction of the PI3K/Akt signaling pathway. These data are consistent with the results of an *in vivo* study by Wang *et al*., in which probiotics could protect β-cells against apoptosis by up-regulating the expression of anti-apoptotic proteins and the PI3K/Akt signaling cascade, and down-regulating the expression of inflammatory factors and pro-apoptotic proteins.^101^ In line with this concept, a recent *in vitro* study by Huang *et al*. looked at the relation between surface components of probiotics and the Akt/mTOR pathway and its inverse association during diabetes onset. Their findings suggest that EPS of *Lactobacillus plantarum* H31 exerts anti-diabetic effects and plays an overall important role in glucose metabolism by up-regulating the expression of Akt-2, AMPK, and human glucose transporter 4 (GLUT-4), by interfering with the pancreas α-amylase activity.^35^

### Cardio vascular disease (CVD)

Several pathways have been implicated in orchestrating the cellular response in CVD. Findings reveal that molecules involved in insulin-like growth hormone (IGF-I)-related survival pathways, including IGF-1 receptor (IGF-IR), IGF-I, *p*-Akt and *p*-PI3K, regulate cardiac survival pathways.^102^ A substantial amount of evidence indicates that an important mechanism in the development of obesity-related heart disease in high-fat diet-fed rats is the dysregulation of anti-apoptotic along with cardiac IGF-I/PI3K/Akt-dependent survival cascades.^103^ Collectively, previous studies have provided strong evidence showing that probiotic supplementation can enhance the activity of survival pathways in obese hearts, suggesting an interplay between IGF1/PI3K/Akt cell survival pathways and some strains of probiotics in the regulation of cardiovascular homeostasis. Along the same line, the *in vivo* effects of probiotics on CVD were investigated by Lin *et al*. and Wang *et al*., who found that oral administration of multi-strain probiotic groups can decrease cardiac apoptosis by up-regulating the IGF-I/PI3K/Akt survival pathway.^103,104^ In parallel studies, it was also reported that high expression of Fas ligand and its receptor Fas are linked to the progression of cardiomyocyte apoptosis. Accordingly, *in vivo* studies documented that heat-killed *Lactobacillus reuteri* GMNL-263 has similar effects on the reduction of cardiomyocyte apoptosis through down-regulating Fas ligand and up-regulating the IGF1R/PI3K/Akt cell survival pathway, thus recovering the myocardial disarray.^50^

### Nonalcoholic fatty liver disease (NAFLD)

One of the hepatic manifestations of MetS is NAFLD, a spectrum of diseases such as nonalcoholic steatohepatitis (NASH), cirrhosis, hepatocellular carcinoma (HCC), and steatosis, characterized by inflammation, increased risk for liver carcinogenesis and fibrosis, and hepatocyte damage/death.^105,106^ Emerging lines of evidence have shown that modulation of autophagy and NF-κB-mediated inflammatory responses are important mechanisms in the pathogenesis of NAFLD.^107^ It has been found that both *Lactobacillus rhamnosus* and its metabolites, along with bone marrow mesenchymal stem cells (BMMSCs), by decreasing autophagy and inflammation through down-regulation of the PI3K/mTOR/NF-κB pathways, have the potential to mitigate alcoholic hepatitis and alleviate its symptoms in *in vivo*.^41^

Some findings imply that high-fructose diets can enhance the prevalence of NAFLD, lipid accumulation, expression of fatty acid synthase (FASN), hepatic weight, but inhibit the expression and phosphorylation of IRS-1/Akt/endothelial nitric oxide synthase (eNOS) signaling intermediates in the liver. On the other hand, these events could be reversed following *Lactobacillus plantarum* supplementation in high-fructose-fed rats, revealing a possible beneficial effect of probiotics on renal and hepatic dysfunction and the prevention of NAFLD.^31^ In this respect, *Clostridium butyricum* MIYAIRI 588 has been used as a butyrate-producing probiotic in NAFLD rats subject to choline-deficient/L-amino acid-defined (CDAA)-diet. In this study *Clostridium butyricum* MIYAIRI 588 could indeed prevent NAFLD progression by reducing the deposition of hepatic lipid and significantly decreasing the content of triglycerides, reversing insulin resistance, and hepatic inflammatory indexes. This preventing effect is a result of substantially elevated levels of expression of proteins contributing to lipolysis or lipogenesis, along with Akt and hepatic AMPK.^53^ In line with this notion, an *in vivo* study documented a reversing effect of *Lactobacillus johnsonii* N6.2 supplementation on the hyperactivation of mTORC1-activating phosphorylation of pAkt in high-fat diet-fed regime and the reduction of metabolic syndrome-associated changes.^59^

## Probiotics and other disorders

With increasing challenges to human health, probiotics have attracted much attention for their capability to modulate the TLR/PI3K/Akt signaling pathway and ameliorate pharyngitis.^66^ TLRs as pattern-recognition receptors (PRRs) can activate innate immunity via sensing invasion of microbial pathogens. Among TLRs, TLR2 orchestrates innate immune and inflammatory responses by recruiting macrophages and modulating PI3K/Akt pathway-dependent autophagy, respectively, when encountering pathogens such as *Staphylococcus aureus*.^108^ Activation of TLR/PI3K/Akt pathway contributes to the activation of the downstream NF-κB signaling cascade, thereby stimulating the expression of pro-inflammatory mediators and the development of inflammatory diseases such as pharyngitis.^109^ Recent *in vitro* and *in vivo* studies recognized that probiotic *Lactobacillus salivarius* AR809 could attenuate the inflammatory response produced by *S. aureus* by elevating autophagic protein level and blocking the TLR/PI3K/Akt/mTOR/NF-κB signaling network.^66^ A substantial amount of evidence demonstrates that administration of probiotics such as *Bifidobacterium animalis* subsp. *lactis* DSM10140 and/or LGG in Caco-2 cells can differentially affect the IL-8 response to *Salmonella* based on the time of administration, in that administration of these probiotics before *Salmonella* infection can enhance the activation of the PI3K/Akt signaling pathway, while it inhibits PI3K/Akt pathway activation after *Salmonella* infection.^44^ As in many other cases, this information is not highly predictive of the overall effect of probiotics and provides an incomplete picture of how they regulate the PI3K/Akt pathway in *Salmonella* infection. Thus, further experimental studies are required to elucidate this point.

Mucosal delivery of probiotics has been the subject of growing interest due to its proven therapeutic effects in inflammatory and allergic disorders.^110^ Development of food hypersensitivity reactions occurs due to production of food protein-specific immunoglobulin E (IgE) and stimulation of basophils or mast cells, as a result of the activation of imbalanced, T helper 2 (Th2)-biased immune responses.^111^ Extensive studies have been conducted to support the efficacy of probiotic microorganisms in decreasing food allergy symptoms via the regulation of mTOR and Treg cells. An *in vivo* study showed that consumption of *Bacillus coagulans* 09.712 up-regulates the transcription factor forkhead box P3 (Foxp3) and down-regulates the phosphorylation of Akt, 4E-BP1, STAT3, serum/glucocorticoid regulated kinase 1 (SGK1) and p70S6K by inhibiting mTOR. Hence, the induction of the anti-inflammatory cytokine IL-10 and of CD4^+^Foxp3^+^ regulatory T cells (Tregs), and, conversely, the inhibition of T helper 17 (Th17) and Th2-predominant can cause the alleviation of food allergic inflammation.^29^ This notion is further confirmed in studies showing that *Bifidobacterium breve* (BbC50sn), by regulating p38MAPK, ERK, and PI3K/Akt signaling pathways, can exert beneficial effects on allergic and inflammatory disorders depending on its interaction with monocyte-derived dendritic cells (DCs), leading to DC-induced IL-10 production and DC maturation, activation, and survival.^58^ Whereas most similar studies are concordant on the beneficial effects of probiotics on alleviation of allergic disorders, further research efforts are required to answer critical questions on the mechanisms underlying definite aspects of human-probiotic relations to fully understand the therapeutic potential of probiotics in allergic and inflammatory disorders.

The process of melanin synthesis called melanogenesis contributes significantly to protection of the skin from UV radiation. Extreme accumulation of melanin causes hyperpigmentation disorders.^112^ Topical depigmentation agents have long been employed in the treatment and/or prevention of these disorders. Notably, it is well documented that the PI3K/Akt signaling cascade in cutaneous stem cells is critically involved in hyperpigmentation disorders and can control their apoptosis and proliferation both in human and murine models.^113^ As a result, this pathway has been considered a suitable target in several types of skin disorders. Studies in B16F10 mouse melanoma cells have provided evidence to suggest that LTA isolated from *Lactobacillus plantarum* can prevent melanogenesis by inhibiting the expression of melanogenic enzymes and cellular activity of tyrosinase, while activating the PI3K/Akt pathway and ERK.^32^ A very recent study has demonstrated that exposure of a cell-free extract of *Enterococcus faecium* L-15 to mouse skin-derived precursor cells (SKPs) could result in improvement of self-renewal and proliferation of SKPs by activation of the PI3K/Akt signaling pathway. Therapies with mesenchymal stem cells (MSCs) have been confirmed to accelerate wound healing of skin and mucosa due to increased tissue regeneration rates.^6^ However, a microecological imbalance can impair the function of MSCs, leading to retarded wound healing. Recent *in vivo* investigations indicate that *Lactobacillus reuteri* extracts can accelerate the wound healing process in the oral mucosa by stimulating the functions of gingiva MSCs (GMSCs). Mechanisms accounting for these effects include the enhancement of active-β-catenin and transforming growth factor β1 (TGFβ1) expression and of PI3K/Akt pathway phosphorylation.^49^ Additional work and more clinical evidence are needed to fully understand whether the effects of probiotics on hyperpigmentation disorders are mediated by an underlying regulation of the PI3K/Akt signaling cascade.

## Conclusion

The PI3K/Akt/mTOR signaling pathway is one of the most important signal transduction pathways that have many biological functions and drives numerous cellular and physiological processes in the body. Extensive research has provided a better knowledge of the molecular mechanisms controlling the PI3K/Akt/mTOR signaling cascades. As reviewed herein, a vast body of evidence demonstrates that aberrant activation and/or dysregulation in the major components of the PI3K/Akt/mTOR signaling pathways are identifiable in different diseases, including most, if not all, human cancers. The last decade has witnessed much progress in our knowledge of the properties of lactic acid bacteria (LAB) as probiotic candidates,^7,^^114^ which include their modulatory functions on specific targets of the PI3K/Akt/mTOR signaling pathway, and their promising effects on infection control. Driven by earlier evidence of their effects on the PI3K/AKT/mTOR axis, research focused on elucidating the molecular mechanisms of various probiotics and their derived metabolites has rapidly gained momentum. Most current efforts are directed toward the characterization of innovative approaches to target this important pathway in the attempt to develop promising and selective treatment options. In this review, we extensively discussed a number of *in vitro* and *in vivo* studies conducted with several strains of probiotics to understand their mechanisms of action on the PI3K/Akt/mTOR network in several disease models. However, despite intensive efforts by many researchers, nearly all of the studies conducted to date have been conducted *in vitro* or in animal models. Thus, definitive evidence for their beneficial effects in human disease is still lacking, and more research needs to be carried out in human subjects and clinical samples. Such studies, and in particular those concentrating on probiotic strains with documented effects on components of the PI3K/Akt/mTOR pathway, would be likely to provide more conclusive outcomes and support further exploration for new therapeutic candidates for the treatment of various, highly prevalent disorders.
